# Population genetic structure of sharpbelly *Hemiculter leucisculus* (Basilesky, 1855) and morphological diversification along climate gradients in China

**DOI:** 10.1002/ece3.7528

**Published:** 2021-05-01

**Authors:** Lihong Wang, Long Zhu, Kui Tang, Mengyu Liu, Xue Xue, Gaoxue Wang, Zaizhao Wang

**Affiliations:** ^1^ College of Animal Science and Technology Northwest A&F University Yangling China

**Keywords:** climate, *Hemiculter leucisculus*, morphological diversification, selective pressure

## Abstract

Sharpbelly *Hemiculter leucisculus* (Basilewski, 1855) is a small, widespread, and native cyprinid fish with prominent habitat suitability and high invasive potential and is becoming the dominant species in freshwater ecosystems under intensified environmental disturbances. But how *H. leucisculus* acclimates to extremely heterogeneous environments remains unclear. In current study, the genetic structure of *H. leucisculus* was analyzed using Bayesian phylogenetic inference, haplotype network, and STRUCTURE base on *cytb* gene across 18 populations spanning 20 degrees of latitude and 18 degrees of longitude in China. The morphological diversification of body size and shape for *H. leucisculus* along the climate gradient was studied. The results showed that the 18 *H. leucisculus* populations were divided into 3 clusters: one cluster mainly from Huanghe River Basin, another cluster mainly from Yangzi River Basin, and H cluster containing Hainan and Beihai populations. The fish from southern populations were deeper bodied while individuals from northern populations were more slender. Inland individuals were more streamlined while coastal individuals were of deeper body. The partial Mantel test predicts that the potential mechanism underlining the intraspecies morphological diversification along climate gradients is primarily the divergent selection pressures among different environments, while genetic variation had less contribution to morphological differentiation. The formation of the Nanling Mountain Range could drive genetic differentiation between Beihai population and those from Yangzi River Basin. The present results highlight strong selective pressures of climate on widespread species and enrich morphological differentiation basis of acclimation for species with high habitat suitability and invasive potential.

## Graphical abstract

Previous studies indicate *Hemiculter leucisculus* has prominent habitat suitability and high invasive potential. This manuscript shows divergent selection pressures among different environments shape morphological diversification for sharpbelly. It predicts that divergent selective pressures are crucial in determining body shape in sharpbelly, while genetic variation has less contribution to morphological differentiation.

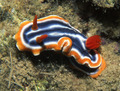

## 1 INTRODUCTION

Understanding environmental factors driving phenotypic diversification along climatic gradients has been a challenging goal in evolutionary biology and biogeography for decades (Kraft et al., 2011; Quesada et al., 2007; Reznick et al., 2001; Riesch et al., 2018). The variation of environmental conditions along climatic gradients including abiotic and biotic factors generates divergent selective regimes which affect phenotypic traits tightly connected to the survival and reproduction of organisms (Culumber et al., 2012). Studies on phenotypic trait variations over large geographical scales can give insights into evolutionary mechanisms (Ouyang et al., 2018).

Inhabiting in distinct ecological environments and being highly plastic body morphology, freshwater fish are considered as perfect systems for investigating the connection between morphology and environmental gradients (Rundle, 2002; Schluter, 1995; Shuai et al., 2018). With multitasking capabilities, the body morphology of fish affects swimming‐related performance including searching, migration, prey capturing, and predator avoidance, also nonswimming performances such as prey processing, suction, burrowing, and crawling (Walker, 2010). The swimming performance variations for fishes usually imply to morphological plasticity (Georgakopoulou et al., 2007; Gerry et al., 2011). Variant environmental factors exert differential selective pressure on the swimming performance of fish. Water velocity gave rise to morphological plasticity on locomotion relevant traits such as fin size and body shape in fish (Senay et al., 2015; Yavno & Fox, 2013). Under high water velocity, a streamlined body shape, slender caudal peduncle, and small fins for fishes will lower drag to facilitate quick swimming (Langerhans & Reznick, 2010; Webb, 1984). On the contrary, stout body and caudal peduncle will improve maneuverability and burst swimming and raise foraging efficiency under low water velocity (Langerhans & Reznick, 2010; Webb, 1984). As a key biotic factor, variation in predator pressure often resulted in morphological plasticity of prey organisms by favoring improved avoidance performance (McPeek, 1997; Nilsson et al., 1995). The mosquitofish populations exposed to predators showed more streamlined body, expanded caudal region, and ventrally positioned eyes compared with predator‐free populations (Arnett & Kinnison, 2017; Langerhans et al., 2004).

Sharpbelly *Hemiculter leucisculus* (Basilewski, 1855) is a small native cyprinid species with little commercial importance. They were reported to be distributed across China, Vietnam, the Korea Peninsula, Mongolia, and the Fareast of Russia (CABI, 2016; Dai & Yang, 2003). They have already invaded Central Asian countries including Kazakhstan, Uzbekistan, and Turkmenistan (Borisova, 1972; Petr & Mitrofanov, 1998; Sal'nikov, 1998), and Western Asia such as Iran, Iraq, and Azerbaijian (Coad & Hussain, 2007; Esmaeili & Gholamifard, 2011; Mustafayev et al., 2015), by accidental introduction in most cases. *H. leucisculus* has been found to feed on eggs and fry of some indigenous fish directly or compete with the juveniles of these fish to the detriment of local ecosystem (CABI, 2016). Dong et al. (2020) projected that *H. leucisculus* is of global habitat suitability and high invasive risk potential with wide distribution over the world except Antarctica. As an ecological generalist (Chen, 2008), *H. leucisculus* was speculated to be of higher invasion potential in habitats with more human disturbance (Dong et al., 2020).

With the intensified environmental disturbance over last few decades, *H. leucisculus* gradually became the dominant species in the fish communities from Yangzi River and Pearl River of China, with the abundance percentage as high as 61% at upper Yangzi River (Gao et al., 2010; Tan et al., 2010). In a native translocation at Erhai Lake in southwestern China, *H. leucisculus,* which initially occurred in 2004, dominated the fish community with abundance percentage as high as 76% by 2012 (Wang et al., 2016). The increasing anthropogenic activities and the subsequent environmental disturbances are characterized by simplified community, little predation, decreased competition, and abundant organic matters, which probably account for the dominant position of *H. leucisculus* with high suitability and broad tolerance. Therefore, *H. leucisculus* could be one of the few beneficiaries of environmental disturbances for wide distribution and become increasingly dominant in freshwater ecosystems (McKinney & Lockwood, 1999). As a widespread species with prominent habitat suitability and high invasive potential, *H. leucisculus* is dominant in diverse fish communities with extremely heterogeneous environments, especially in the context of intensified anthropogenic activities. However, it is still far from clear how *H. leucisculus* acclimate to extremely heterogeneous environments. As a proxy for a species' ecological role in a community, the external morphology was an effective tool for assessing the phenotypic differentiation (Azzurro et al., 2014). Here, we examine morphological variation in eighteen *H. leucisculus* populations along a latitudinal gradient in basins of Huanghe River, Yangzi River, Nanliujiang River, Qiantang River, and Changhuajiang River of China. The goal of this study is to assess the effects of climate gradient on morphological divergence for the species with high habitat suitability.

## 2 MATERIALS AND METHODS

### 2.1 Sampling sites and climatic data

**FIGURE 1:**
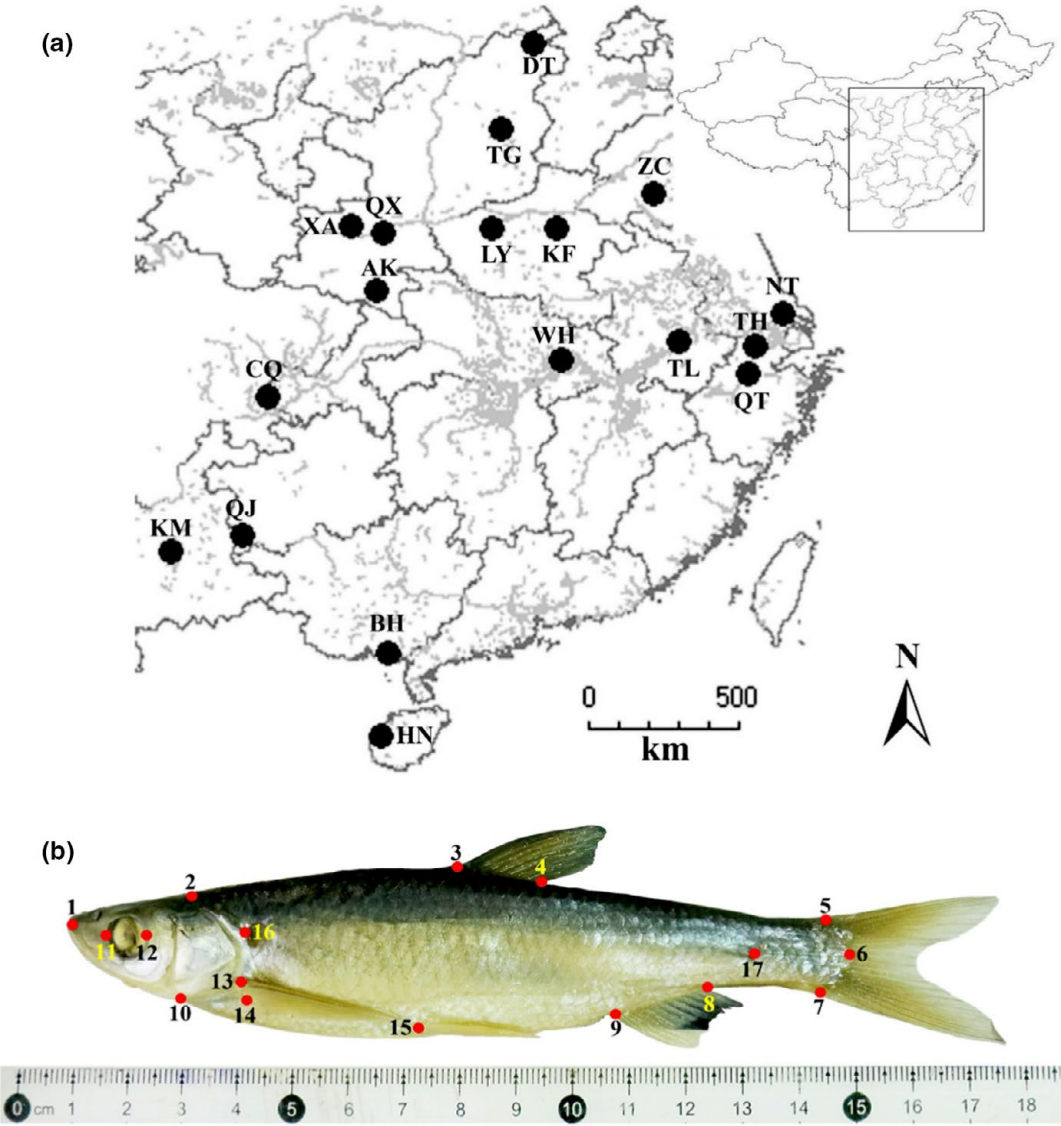
Sampling locations and morphological landmarks for *Hemiculter leucisculus*. (a) The sampling locations across China from which *H. leucisculus* were collected. (b) *H. leucisculus* was collected in Datong April 2017. Dots denote 15 landmarks for morphometric analysis and 2 additional landmarks (No. 16 and 17) for unbending procedure in Procrustes analysis

Sharpbelly *H. leucisculus* samples were identified morphologically following taxonomical classification of Chen (1998). We collected sharpbelly with 12–14 cm length (*n* = 545) from 18 locations (Figure 1a) using gill nets with an inner mesh of 30 mm and an outer mesh of 110 mm in spring of 2016 and 2018. As shown in Table S1, sampling sites of Chongqing (CQ), Wuhan (WH), Ankang (AK), Tongling (TL), Taihu (TH), Nantong (NT), Kunming (KM), and Qujing (QJ) are located at Yangtze River Basin. Sites of Qianxian (QX), Xi'an (XA), Datong (DT), Taigu (TG), Zoucheng (ZC), Kaifeng (KF), and Luoyang (LY) are located at the Huanghe River Basin. The site of Beihai (BH) is located at the Nanliujiang River Basin. Qiantangjing (QT) population was collected from Qiantang River Basin. The Hainan (HN) population was sampled at the Changhuajiang River Basin in Hainan Island. Upon collection, the fish were immediately euthanized using clove oil and preserved in 95% ethanol.

The climatic data from 1981 to 2010 were downloaded from the Chinese meteorological data network (http://data.cma.cn/). The data included mean annual temperatures, maximum temperatures of the warmest month, minimum temperatures of the coldest month, annual temperature differences (by subtracting the minimum monthly temperature from the maximum monthly temperature), and annual precipitation to provide site‐specific climatic information (Table S2). Altitude and distance to the sea were obtained from Google Earth (http://earth.google.com/) (Table S2). Those variables were condensed by principal component analysis (PCA), resulting in two principal components (PCs) with eigenvalues >1 that explained 83.230% of the variation (Table 1). PC1 represented the gradient from northern to southern sites (latitudinal variation) with higher mean annual temperatures, higher minimum temperatures of the coldest month, higher annual precipitation, higher maximum temperatures of the warmest month, and lower annual temperature differences at southern sites. PC2 represented gradual changes from coastal toward inland sites (longitudinal variation) with higher altitude, longer distance to sea, and lower temperature of the warmest month. The two climate‐related PCs (cPCs) were used as covariates in subsequent analyses to test phenotypic divergence along climatic gradients.

**TABLE 1:** Results of principal component analyses on environmental factors across sampling sites^a^

	Principal component1	Principal component2
Eigenvalue	3.654	2.172
Variance explained [%]	52.200	31.030
Mean annual temperature	**0.941**	−0.129
Maximum temperature of the warmest month	−0.213	**−0.807**
Minimum temperature of the coldest month	**0.964**	0.064
Annual temperature difference	**−0.861**	0.233
Annual precipitation	**0.957**	−0.110
Altitude	−0.281	**0.944**
Distance to the sea	−0.24	**0.736**

^a^ Shown are axis loadings for PCs with eigenvalues ≥ 1.0. and axis loadings ≥ |0.6| are highlighted in bold font.

### 2.2 DNA extraction, amplification, and sequencing

The total genomic DNA was extracted from pectoral fin tissue of specimens preserved in 95% ethanol using the EasyPure Genomic DNA Kit (Beijing TransGen Biotech, Beijing, China). The mitochondrial cytochrome b gene (*cytb*) was used as molecular marker (Xiao et al., 2001). The primer sequences for *cytb* amplification are 5′GACTTGAAAAACCACCGTTG 3′ (forward) and 5′ CTCCGATCTCCGGA TTACAAGAC 3′ (reverse). The PCR amplification was performed in a final volume of 50 µl reaction mix with 25 µl 2 × Taq MasterMix (CWBIO, Beijing, China), 4 µl primer mix, 8 µl template DNA, and RNase‐free water. Thermocycling condition was as follows: initial denaturation at 94°C for 3 min, followed by 35 cycles of 94°C for 30 s, 58° for 30 s, 72°C for 30 s, and a final elongation step at 72°C for 10 min. The PCR products were isolated via gel electrophoresis and purified with the EasyPure Quick Gel Extraction Kit (TransGen Biotech, Beijing, China) and then sequenced by Tsingke Corporation (Xi'an, China).

The *cytb* gene was obtained from 365 individuals of *H. leucisculus* containing 144 polymorphic sites and 108 parsimony‐informative sites. A total of 172 unique haplotypes were defined. All sequences analyzed in this study have been deposited in GenBank under Accession No. MW006099‐MW006463.

### 2.3 Body shape analysis

The geometric morphometric method was used to characterize the body shape (Rohlf & Marcus, 1993). The left lateral photographs were taken in a paraffin‐coated dish alongside a piece of scale ruler using a Canon EOS 760D camera (Canon Inc., Tokyo, Japan). Specimens without significant bending, deformities, or breeding characteristics were chosen to photograph (*n* = 432, 20–31 individuals per population). Photographs were transformed to tps format using tpsUtil v. 1.75. Total 17 landmarks (Figure 1b) were determined following the established protocols by Riesch et al. (2016). The 17 landmarks were digitized by tpsDig2 v. 2.31 for each fish. These landmarks are (1) tip of the upper jaw; (2) the posterodorsal tip of the supraoccipital crest; (3) anterior and (4) posterior insertions of the dorsal fin; (5) the dorsal, (6) central, and (7) ventral insertions of the caudal fin; (8) the posterior and (9) anterior insertions of the anal fin; (10) the bottom of the head where the operculum breaks away from the body outline; (11) the anterior and (12) posterior margin of the eye; (13) the dorsal and (14) ventral insertions of the pectoral fin; (15) anterior junction of the pelvic fin; (16) the intersection of the lateral line with the posterior margin of the operculum; and (17) the intersection of the lateral bending upward with the center of the caudal peduncle. Landmarks provided adequate coverage of the lateral body contour. To correct for bending effects, the “Unbend specimens” function in tpsUtil v. 1.75 was performed with landmarks 1, 6, 16, and 17. A full Procrustes fit procedure was performed using the software MorphoJ v. 1.06d which superimposes shape coordinates in a linear tangent space and automatically excludes variation that is not caused by true shape variation (i.e., translation, scaling, and rotation effects). No individual with outliers was found after the unbending analysis. After extracting shape information, a factor reduction procedure was performed in MorphoJ to reduce data dimensionality (Klingenberg, 2011). The retained ten relative warps (RWs) accounted for 84.656% of the total morphological variance (Table 2).

**TABLE 2:** The morphological relative warps and their respective variances accounted for cumulative variance

RWs	Eigenvalues	Variance %	Cumulative %
1	0.00026153	15.829	15.829
2	0.00025097	15.19	31.019
3	0.00019937	12.066	43.085
4	0.00018403	11.138	54.223
5	0.00012741	7.711	61.935
6	0.00010353	6.266	68.2
7	0.00008838	5.349	73.55
8	0.00008061	4.879	78.429
9	0.00005586	3.381	81.81
10	0.00004703	2.846	84.656

### 2.4 Statistical analysis

#### 2.4.1 Population genetic structure

Total and net genetic divergences between lineages were calculated using Kimura 2‐parameter distances in Mega v. 6.0. Bayesian clustering analysis (Pritchard et al., 2009) was performed to calculate individual assignment probabilities (*Q*‐values) to varying numbers of genetically distinct clusters (*K*). For each value of *K* = 1–10, ten independent iterations were run using the assumed admixture model with a burn‐in period of 250,000 generations, followed by a sampling phase of 750,000 Markov Chain Monte Carlo (MCMC) iterations. The uppermost level of population differentiation was detected using the web‐based tool STRUCTURE HARVESTER v. 0.6.94 (Evanno et al., 2005). The grouping of the *H. leucisculus* populations was determined according to the most likely number of genetic clusters K calculated by STRUCTURE HARVESTER and the clade from BI tree.

In addition, the phylogenetic relationships among haplotypes were inferred by the Bayesian phylogenetic inference (BI) of MrBayes v. 3.2.2 (Ronquist et al., 2012). Alignments for each locus were generated with Muscle in Mega v. 6.0 using default parameters. Following the Akaike information criterion, nucleotide substitution models (GTR + I + G) were selected as the best‐fit model of sequence evolution to construct BI tree in the software jModelTest ver. 2.1.10 (Darriba et al., 2012). Four *cytb* genes of *H. bleekeri* including one from GenBank (GenBank no. KF029693) and three sequences of *H. bleekeri* sampled at Changshu, Ankang, and Zhenjiang in Yangzi River Basin (named CS26, AK098, and ZJ009 respectively) were used as outgroups. Thirteen additional *cytb* sequences of *H. leucisculus* randomly picked from GenBank (Table S3) were incorporated in phylogenetic tree construction to validate species identity and enrich geographical locations. Bayesian posterior probabilities were estimated, from two runs with four chains of 10,000,000 generations, sampling trees every 100 generations, with the initial 25% of trees discarded as burn‐in after stationarity was reached. Phylogenetic networks were computed with a median‐joining method in the software NETWORK 5.0 (Bandelt et al., 1999).

The pairwise *F_ST_* values were derived using DnaSP (Table S4) (Rozas et al., 2017). To clarify genetic diversity partitions between populations and each group, a *F_ST_*‐based analysis of molecular variance (AMOVA) was conducted in Arlequin 3.5 (Excoffier & Lischer, 2010).

#### 2.4.2 Morphological variation among populations

Body size is represented by centroid size (CS), the square root of the summed squared distances from each landmark to the configuration centroid, and calculated using MorphoJ v. 1.06d. Log_10_‐transformed Centroid size (Log_10_CS) was used in subsequent analysis. ANCOVA was performed with Log_10_CS as dependent variables and the two climate‐related PCs (cPCs, see above) as covariates. In addition, the interaction terms of both cPCs were treated as covariates. To evaluate the relative importance of each term, the effective size was estimated by calculating Wilk's partial eta squared ($\eta_{p}^{2}$). To visualize significant interaction effects, the data were divided into inland (cPC2 ≥ median) and coastal populations (cPC2 < median) to depict variation along cPC1 (latitudinal variation). The data were also split based on median value of cPC1 to depict the variation along cPC2.

To remove the effect of allometry, the residuals were held as morphology‐related PCs (mPCs) by regressing RWs against centroid size in MorphoJ v. 1.06d. To assess the extent of divergence along climatic gradients, multivariate analysis of covariance (MANCOVA) was employed with mPCs as dependent variables and the two cPCs and the interaction terms of both cPCs as covariates. To identify the sources of variation in case of significant model terms, post hoc ANCOVAs of the exact same structure were run as the final retained MANCOVA model. Wilk's $\eta_{p}^{2}$ was calculated to estimate the effect sizes. All statistical analyses unless mentioned otherwise were performed using SPSS 20.0.

The pairwise Mahalanobis distances for body shape between *H. leucisculus* populations were calculated using MorphoJ v. 1.06d. The pairwise climate Mahalanobis distances between populations were based on cPC1 and cPC2 using StatMatch package in R (D'Orazio, 2015). To disentangle effects of genetic variation and environmental differentiation on morphological divergence among populations, partial correlation statistics was computed on the matrixes of pairwise morphological Mahalanobis distances (Table S5), climate Mahalanobis distances (Table S6), and *F_ST_*s using partial Mantel test in the R packages *vegan* (Oksanen et al., 2012).

## 3 RESULTS

### 3.1 Population genetic structure

**FIGURE 2:**
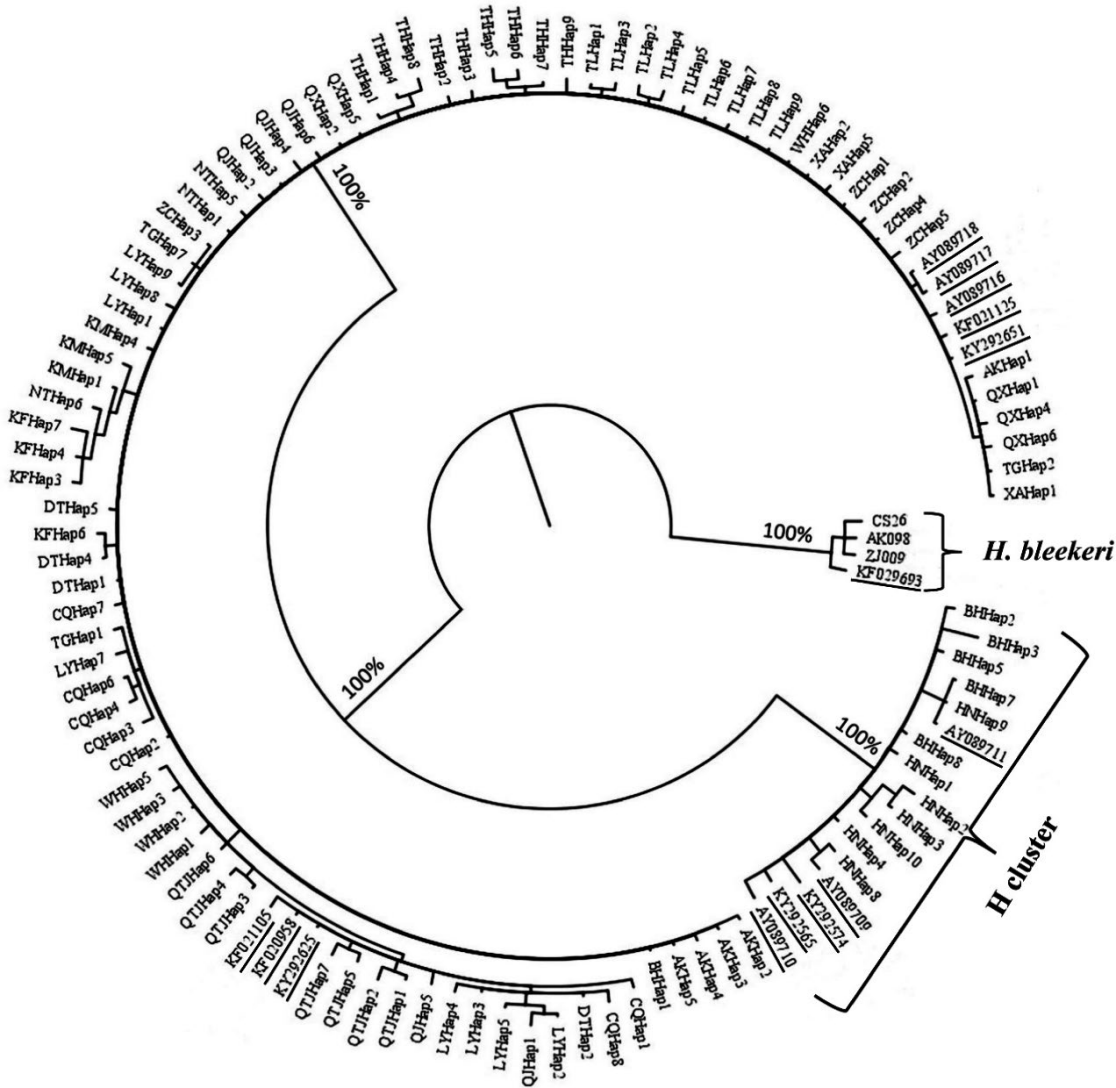
Phylogenetic tree based on Bayesian inference showing the relationships among *Hemiculter leucisculus* populations using *Cytb*. Values on branches indicate Bayesian posterior probabilities. Sequences download from GenBank are underlined. H cluster contains haplotypes from Hainan population, Beihai population, and 5 GenBank *cytb* sequences (3 from Pearl River Basin and 2 from Hainan Island)

**FIGURE 3:**
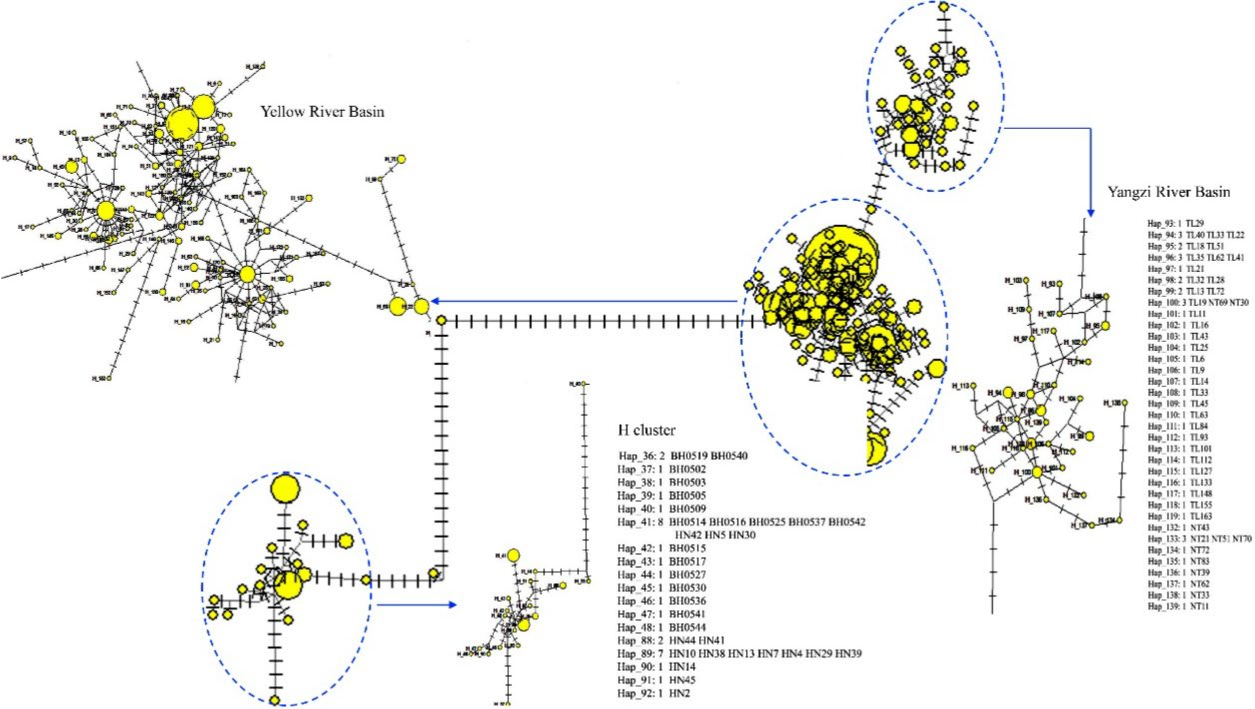
Median‐joining network of *cytb* haplotypes. Each yellow circle represents different haplotypes, scaled according to its frequency in the entire sample. To indicate mutation steps between haploptypes, lines with scales are shown. H cluster contains haplotypes from Beihai and Hainan populations. Yellow River Basin specifies haplotypes from populations from Yellow River Basin. Yangzi River Basin specifies haplotypes from populations from Yangzi River Basin

Based on *cytb* sequences, the BI tree showed two distinct clades (Figure 2). The Cluster H contained all haplotypes of Hainan and Beihai populations and six *cytb* sequences registered in GenBank including four from Guangxi Province and two from Hainan Island. The other clade contained haplotypes from Yellow River, Yangtze River, and Qiantang River Basins, seven GenBank *cytb* sequences including four from Yangtze River, one from Chiu‐lung River, Qiantang River, and Min River Basins, respectively. Consistent with the BI tree, the median‐joining network analyses for *cytb* also showed two obviously separated clades (Figure 3). All haplotypes from Beihai population (belonging to Pearl River Basin) and Hainan population are contained in H cluster. The remaining haplotypes were grouped into two subclusters, consisting of haplotypes from *H. leucisculus* populations from Yangzi River Basin and Yellow River Basin, respectively.

**FIGURE 4:**
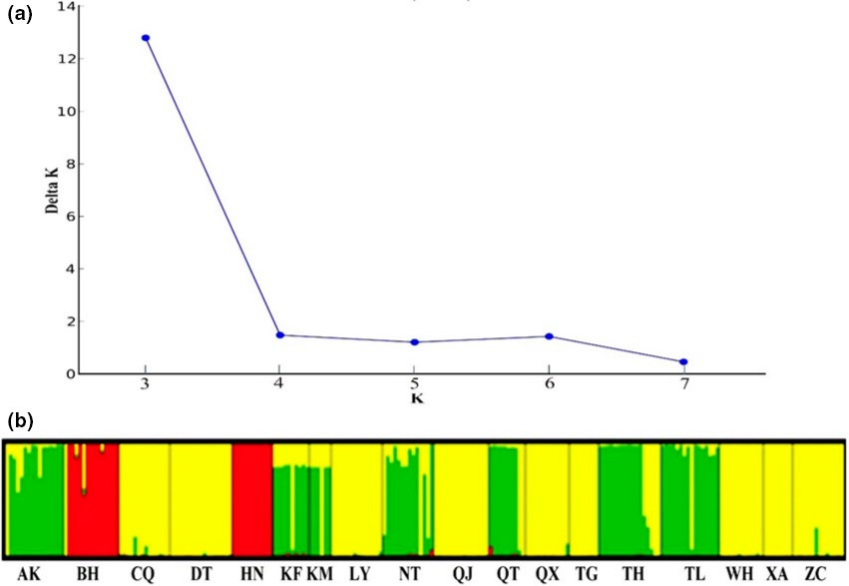
Genetic structure among 18 populations. (a) *K* = 3 was the most likely number of genetically cluster among the 18 populations using STRUCTURE HARVESTER v. 0.6.94 (Evanno et al., 2005). (b) Individual assignment to three genetically distinct clusters using STRUCTURE v. 2.3.4. Assignment likelihood of each individual is shown as a vertical bar (see Table S1 for population codes)

STRUCTURE analysis indicated that *K* = 3 is the most likely number of genetically distinct clades (Figure 4a). The STRUCTURE result showed that 18 *H. leucisculus* populations were divided into distinct three clusters. The first cluster contained Beihai (BH) and Hainan (HN) populations. Seven populations containing Ankang (AK), Kaifeng (KF), Kunming (KM), Nantong (NT), Qiantangjiang (QT), Taihu (TH), and Tongling (TL) formed the second cluster. The remaining nine populations including Chongqing (CQ), Datong (DT), Luoyang (LY), Qujing (QJ), Qianxian (QX), Taigu (TG), Wuhan (WH), Xi'an (XA), and Zoucheng (ZC) composed the third cluster (Figure 4b).

As shown in Table S4, 149 out of 153 pairwise *F_ST_* values were statistically significant ranging from 0.05410 (between Luoyang and Zoucheng, *p* = .045) to 0.96023 (between Xian and Hainan, *p* < .001). There were varying degrees of genetic differentiation between populations, ranging from virtual panmixis (e.g., *F_ST_* = 0.00986, between Qianxian and Luoyang), moderate genetic differentiation (e.g., *F_ST_* = 0.14236, between Chongqing and Wuhan), higher genetic differentiation (e.g., *F_ST_* = 0.234548, between Luoyang and Xi'an) to very high genetic differentiation (e.g., *F_ST_* = 0.96023, between Hainan and Xi'an).

AMOVA showed the overall *F_ST_* was 0.78731(Table 3). AMOVA result demonstrated that 54.88% of the genetic variation was attributed to differentiation among groups (*p* < .001), 23.86% of variation was accounted for among populations within groups, and 21.27% was accounted for within populations, suggesting that *H. leucisculus* populations were highly structured geographically.

**TABLE 3:** Results of an analysis of molecular variance (AMOVA) for three grouping options of *Hemiculter leucisculus* populations based on *cytb*

Source of variation	*df*	Sum of square	Variance component	Percentage of variation
Among groups	2	1,433.749	6.17955	54.88
Among populations within groups	15	855.321	2.68632	23.86
Within populations	347	840.662	2.39505	21.27
Total	364	3,129.732	11.26092	
	*F_ST_* : 0.78731		*p* < .001	

### 3.2 Body size divergence

**FIGURE 5:**
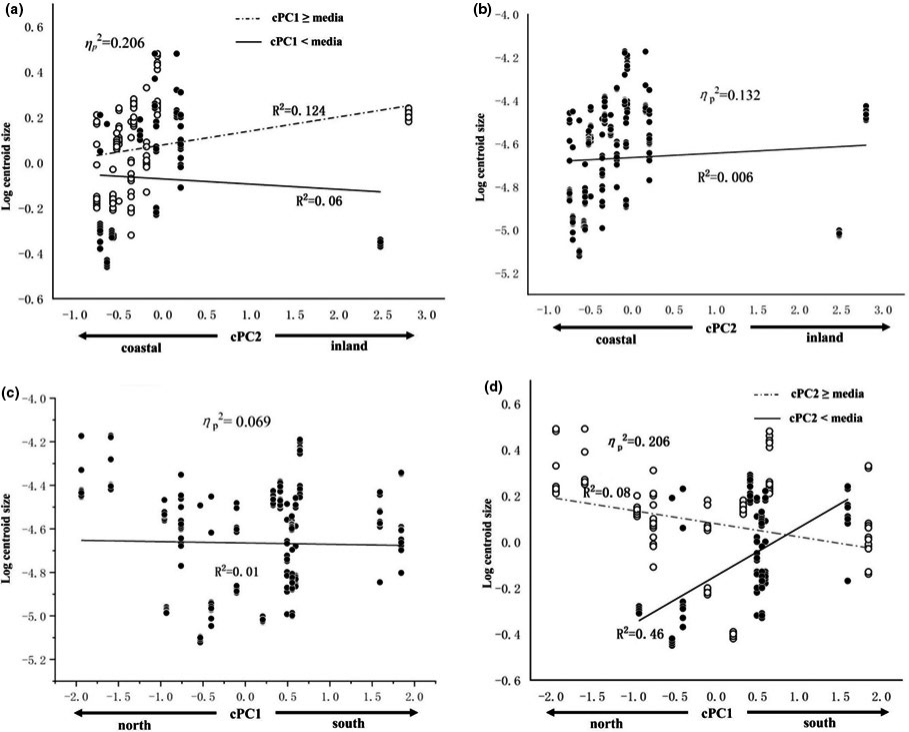
Scatter plot and linear fit of body size (Log_10_ centroid size) along climatic gradients. Visualization of the main effect and interaction of both cPCs on body size. (a) Individuals from south sites (cPC1 ≥ median) showed a trend of increasing Log_10_CS along cPC2 while north populations (cPC1 < median) showed the opposite pattern. (b) Increased trend of body size as cPC2 increased. (c) Individuals from inland sites (cPC2 ≥ median) a trend of decreasing log_10_CS along cPC1 while or coastal populations (cPC2 < median) showed the opposite trend. (d) Log_10_ centroid size showed decreases as cPC1 increased

The divergence of centroid size which represents body size of fish along environmental gradients is shown in Table 4. The ANCOVA result reflected that body size was affected by cPCs and their interactions. According to $\eta_{p}^{2}$ magnitude, the interaction of cPC1 and cPC2 showed the strongest effect ($\eta_{p}^{2}$ = 0.206), followed by cPC2 ($\eta_{p}^{2}$ = 0.132) and cPC1 ($\eta_{p}^{2}$ = 0.069). For the effects of interaction of cPC1 and cPC2, along cPC2, the fish in more southern sites (cPC1 ≥ median, low latitude) were getting bigger from coastal to inland (*R* ^2^ = 0.124; Figure 5a), while a tendency toward a reversed pattern was observed in more northward populations (*R* ^2^ = 0.06; Figure 5a). The main effect of cPC2 showed that body size of *H. leucisculus* became bigger from coast toward inland (*R* ^2^ = 0.006; Figure 5b). Along cPC1, for fish from inland (cPC2 ≥ median, more distance to sea and higher altitude), the northern fish were bigger than the southern fish (*R* ^2^ = 0.08; Figure 5c), while fish from coastal (cPC2 < median) appeared reversed pattern (*R* ^2^ = 0.46; Figure 5c). The main effect of cPC1 on body size reflects that the fish of northern populations were bigger than southern populations (*R* ^2^ = 0.01; Figure 5d), suggesting the body size increase with increasing latitude.

**TABLE 4:** Results from ANCOVA on Log centroid size of *Hemiculter leucisculus* populations

Model	Source	*df*	*F*	*p*	$\eta_{p}^{2}$
Log centroid size	cPC1	1	26.191	<.001	0.069
	cPC2	1	53.378	<.001	0.132
	cPC1 × cPC2	1	90.916	<.001	0.206
	Error	428			

### 3.3 Body shape variation

**FIGURE 6:**
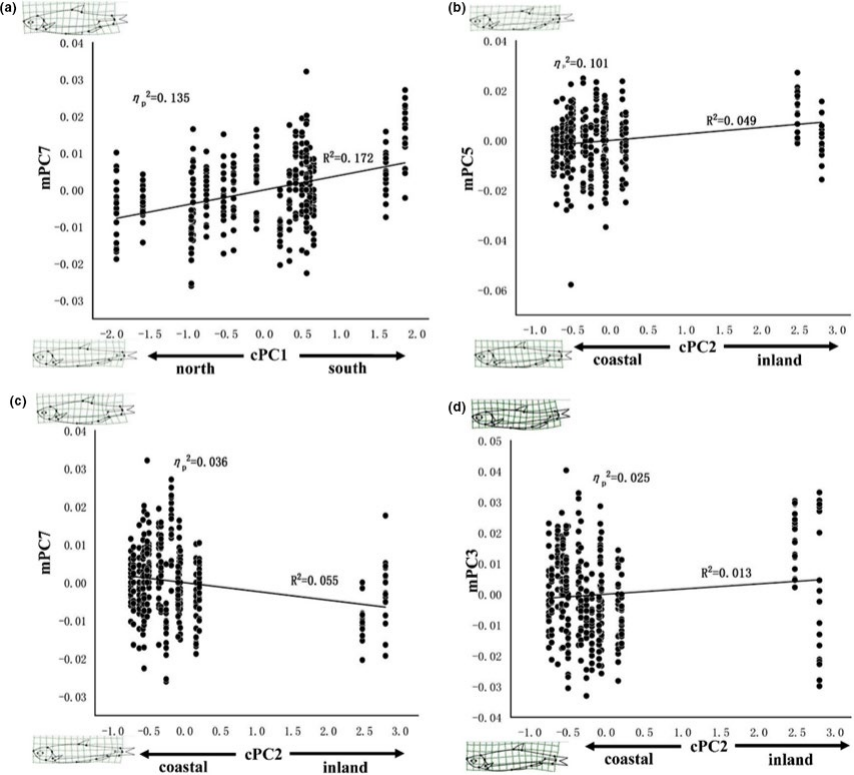
Scatter plot and linear fit of body shape along climatic gradients. Visualization of the main effects of both climatic PCs on morphology‐related PCs. (a) mPC7 change along climatic PC1 (cPC1); (b) mPC5 change along cPC2; (c) mPC7 change along cPC2; (d) mPC3 change along cPC2

MANCOVA results showed all covariates had significant effects on body shape (Table 5). The cPC2 showed the strongest effect ($\eta_{p}^{2}$ = 0.218) on body shape, followed by cPC1 ($\eta_{p}^{2}$ **= 0.205) and the interaction of cPC1 and cPC2 ($\eta_{p}^{2}$ **= 0.134). The post hoc ANCOVA data showed the main effects of 2 cPCs and interaction effects of them on every single mPCs (Table 6). The main effects of cPC1 and cPC2 on morphology‐related PCs were displayed in Figure 6. The cPC1 exerted strong positive influence on mPC7 ($\eta_{p}^{2}$ = 0.135; *R* ^2^ = 0.172; Figure 6a), reflecting that the southern fish had shorter caudal peduncles and deeper bodies. The cPC2 had positive effect on mPC5 ($\eta_{p}^{2}$ = 0.101), suggesting that the inland fish were longer and had more posteriorly positioned dorsal fins compared with coastal populations (*R* ^2^ = 0.049; Figure 6b). However, it exerted negative influence on mPC7 ($\eta_{p}^{2}$ = 0.036), indicating inland fish were slightly slender compared with coastal fish (*R* ^2^ = 0.055; Figure 6c). The cPC2's weak effect on mPC3 ($\eta_{p}^{2}$ = 0.025) suggests that inland fish tend to have marginally longer anal fin and larger eye than coastal fish (*R* ^2^ = 0.013; Figure 6d). In brief, the main effects of cPC2 on body shape of inland fish or high altitude populations tend to have more slender body, posterior positioned dorsal fins, shorter caudal peduncles, and longer anal fins.

**TABLE 5:** MANCOVA on morphological traits of *Hemiculter leucisculus*

Model	Source	*df*	*F*	*p*	$\eta_{p}^{2}$
Morphology‐related PCs	cPC1	10	8.780	<.001	0.205
	cPC2	10	9.487	<.001	0.218
	cPC1 × cPC2	10	5.259	<.001	0.134
	Error	419			

**TABLE 6:** Tests of between‐subjects effects of body shape based on ANCOVA*

Model	Source	Dependent Variable	*df*	*F*	*p*	$\eta_{p}^{2}$
Body shape‐related PCs	cPC1	mPC1	1	1.917	.167	0.005
		**mPC2**	**1**	**4.795**	**.029**	**0.014**
		mPC3	1	0.487	.486	0.001
		mPC4	1	1.953	.163	0.006
		mPC5	1	2.040	.154	0.006
		**mPC6**	**1**	**4.339**	**.038**	**0.012**
		**mPC7**	**1**	**54.767**	**<.001**	**0.135**
		mPC8	1	0.578	.447	0.002
		mPC9	1	1.085	.298	0.003
		**mPC10**	**1**	**11.721**	**.001**	**0.032**
	cPC2	mPC1	1	1.490	.223	0.004
		mPC2	1	2.231	.136	0.006
		**mPC3**	**1**	**9.008**	**.003**	**0.025**
		**mPC4**	**1**	**5.421**	**.020**	**0.015**
		**mPC5**	**1**	**39.172**	**<.001**	**0.101**
		mPC6	1	0.153	.696	0.000
		**mPC7**	**1**	**13.202**	**<.001**	**0.036**
		mPC8	1	3.743	.054	0.011
		mPC9	1	0.028	.867	0.000
		mPC10	1	1.786	.182	0.005
	cPC1 × cPC2	mPC1	1	3.306	.070	0.009
		**mPC2**	**1**	**8.318**	**.004**	**0.023**
		mPC3	1	0.886	.347	0.003
		mPC4	1	3.712	.055	0.010
		**mPC5**	**1**	**18.264**	**<.001**	**0.050**
		mPC6	1	2.886	.090	0.008
		mPC7	1	0.013	.910	0.000
		mPC8	1	2.386	.123	0.007
		mPC9	1	0.510	.475	0.001
		**mPC10**	**1**	**10.653**	**.001**	**0.030**

* Statistically significant effects (*p* < .05) are highlighted in bold font.

**FIGURE 7:**
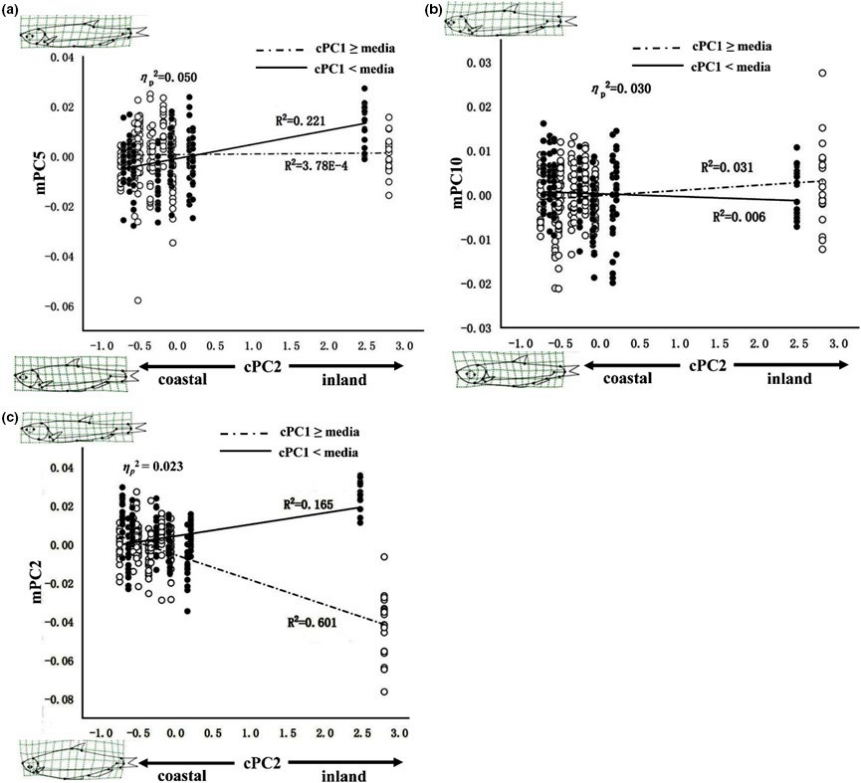
Variant effects of interaction between cPC1 and cPC2 on body shape along cPC2. (a) Effect of interaction between cPC1 and cPC2 on mPC5; (b) the interaction effect on mPC10; (c) the interaction effect on mPC2

**FIGURE 8:**
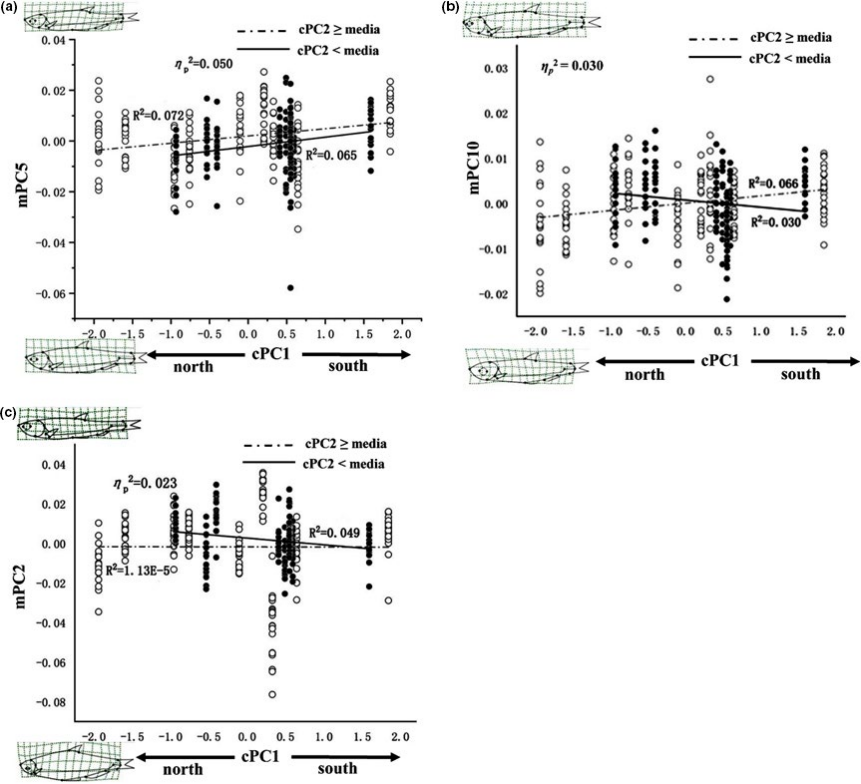
Variant effects of interaction between cPC1 and cPC2 on body shape along cPC1. (a) Effect of interaction between cPC1 and cPC2 on mPC5; (b) the interaction effect on mPC10; (c) the interaction effect on mPC2

The interaction effects between cPC2 and cPC1 on mPCs along cPC2 are visualized in Figure 7. The interaction positively influenced mPC5 ($\eta_{p}^{2}$ = 0.050). In northern populations (cPC1 < median), the inland fish develop more posteriorly positioned dorsal fins and shorter caudal peduncles compared with coastal fish (*R* ^2^ = 0.221; Figure 7a). While for southern populations (cPC1 ≥ median), the interaction has no obvious effect on mPC5. In southern populations, the slight positive influence of the 2 cPC's interaction on mPC10 ($\eta_{p}^{2}$ = 0.030, *R* ^2^ = 0.031; Figure 7b), suggesting that inland fish develop smaller head than coastal fish, while fish of northern populations had no clear variation tendency. For fish from southern populations, the interaction's negative effect on mPC2 ($\eta_{p}^{2}$ = 0.023, *R* ^2^ = 0.601; Figure 7c) suggests that inland fish tend to have deeper body, smaller head, and shorter caudal peduncles compared with coastal fish, while the opposite trend was observed in northern populations (*R* ^2^ = 0.165; Figure 7c). The alternative way depicting variation along cPC1, while splitting the data based on median values of cPC2, is shown in Figure 8. For both inland (cPC2 ≥ median) and coastal (cPC2 < median) populations, the slight positive influence of interaction on mPC5 ($\eta_{p}^{2}$ = 0.050, *R* ^2^ = 0.072, and 0.065; Figure 8a) indicates southern fish tend to be slender and with posteriorly placed dorsal fin compared to northern fish. For inland populations, the weak interaction effect on mPC10 ($\eta_{p}^{2}$= 0.030, *R* ^2^ = 0.066; Figure 8b) suggests the southern fish have a tendency to be with smaller head, while the coastal population is on the opposite. For coastal populations, the slight negative correlation between interaction effect and mPC2 ($\eta_{p}^{2}$ = 0.023, *R* ^2^ = 0.049; Figure 8c) shows southern fish have deeper body and shorter caudal peduncle compared to northern fish.

### 3.4 Association of morphological variation with climatic and genetic distances

The Mantel test results showed that the pairwise morphological Mahalanobis distances had significant correlation with between‐population climatic Mahalanobis distances (*r* = 0.4304, *p* = .005; Table 7). After controlling for genetic distances as a confounding variable using partial Mantel test, there still existed significant correlation between morphological distances and climatic distance (*r* = 0.3681; *p* = .024). In spite of the significant correlation between pairwise morphological distances and F*_ST_*s estimated by Mantel test (*r* = 0.3638, *p* = .016), the weighted evaluation result of partial Mantel test showed that the correlation was not significant (*r* = 0.2818, *p* = .067; Table 7).

**TABLE 7:** The Mantel test and partial Mantel test results between pairwise morphological Mahalanobis distances and climatic Mahalanobis distances, morphological Mahalanobis distances, and *F_ST_*

	Morphological distance				
	Mantel test		Partial Mantel test		
	*r*	*p*	Controlled factor	*r*	*p*
Climatic distance	0.4304	.005	*F_ST_*	0.3681	.024
*F_ST_*	0.3638	.016	Climatic distance	0.2818	.067

## 4 DISCUSSION

In this study, both the BI tree and haplotype network based on *cytb* gene affirm a separate cluster (H cluster) from Hainan sampling site in Changhuajiang River and Beihai sampling site in Nanliujiang River Basin. The STRUCTURE data also support the grouping of Beihai and Hainan populations, which reflects there exist potential barriers to gene flow between this cluster and the remaining populations. All the current data suggest the high genetic differentiation between the H cluster and the others. It could indicate ongoing speciation in *Hemiculter* genus in Nanliujiang River Basin and Hainan Island.

The vicariant events should be conducive to hypothesize that the specific population genetic structuring patterns are consequence of mountain range uplift and sea level fluctuation due to glacial cycles. Investigation on phylogeography of *Prochilodus lineatus*, a freshwater fish widely distributed in the major rivers of South America, explained some geological and geographical events occurred millions of years ago (Sivasundar et al., 2001). Study on zoogeographical division in Pearl River Basin inferred the formation of Nanling Mountain Range, the watershed between Yangtze River and Pearl River, could promote the population differentiation of fish fauna (Chen et al., 1986). The Pearl River is located in subtropical karst region with complex environment, potentially leading to fairly active speciation. It should be the main cause of the difference between ichthyofauna of Yangtze River and Pearl River (Chen et al., 1986). The Nanliujiang River Basin, situating west of Pearl River Basin, also separates with Yangzi River Basin by Nanling Mountain Range. Therefore, it is hypothesized that the evolution and genetic structuring of fish fauna from Nanliujiang River Basin probably parallel with that from Pearl River Basin. The deep genetic divergence between *H. leucisculus* population of Beihai (BH), located at Nanliujiang River drainage, and populations from Yangtze River Basin, presumedly suggests the discrepant fish fauna between Nanliujiang River Basin and Yangzi River Basin. However, in both sides of Alps, the gene flow of bullheads *Cotttus ferrugineus* was unusually not impeded by this geographic barrier (Slechtova et al., 2004). This is due to the cold‐adapted bullheads are able to colonize the highest stretches of water and can disperse via stream capture.

The cluster H consists of HN population from Changhuajiang River Basin at Hainan Island and BH population from Beihai located in Nanliujiang River drainage. Although separated by Qiongzhou Strait, the two populations share fairly high genetic homogeneity. In addition, the NJ morphological tree also showed the two populations got together. According to Zhao and Huang (1978), most of the continental shelves of the Yellow Sea, East Sea, and South Sea were destitute of seawater 17,000–18,000 years ago. The Chinese mainland extended hundreds of kilometers eastward and southward connecting with Taiwan and Hainan Island. In eastern China, the sea level was lower about 120 m in the last glacial maximum (Rohling et al., 1998; Yang, 1985). During the last glaciation (from 14,000 years ago to the beginning of Holocene), a great deal of ice in the northern hemisphere melts rapidly, which led to a general huge rise of sea level. About 7,000 years ago, the sea level rise gradually stopped as the global continental ice flow had disappeared, except for Antarctica and Greenland. So the separation of Hainan Island and the mainland was between 7,000 and 14,000 years ago. Considering that, it is not surprising that the genetic distance between HN and BH populations is small (the pairwise *F*_ST_ = 0.07 between HN and BH).

The relationship of *H. leucisculus* morphology and latitude (Figure 5e) suggests that fish of southern populations are deeper bodied while individuals from north populations tend to be slender. For *Galaxias platei* populations in the southern Andes, latitude also correlated with the morphological variation including head dimension, caudal shape, and fin length (Milano et al., 2006). Along the latitudinal gradient of Patagonia, catfish *Hatcheria macraei* showed morphological differentiation such as caudal peduncle depth, fin length, and numbers of vertebral and fin ray (Chiarello‐Sosa et al., 2018). So the current data suggest the much broad range of temperature regimes along with latitude could partially account for the morphological differentiation of *H. leucisculus* populations. The body shape of *Dicentrarchus labrax* was apt to be more slender at lower temperature during early life stage (Georgakopoulou et al., 2007), being consistent with the body shape variation of *H. leucisculus* that northern fish are thinner than southern fish in this study.

The correlation with cPC2 and morphology‐related PCs (mPC5 and mPC7) suggests that individuals from inland populations are more streamlined while fish of coastal populations are of deeper body. It is concurrent with fishes in the Pearl River, in which high‐elevation individuals were more narrow‐bodied (Shuai et al., 2018). The high elevation in inland implies to lower water temperature and higher flow velocity. Intraspecific morphological divergence accounting for hydrodynamic conditions is widespread in fishes (Haas et al., 2010; Langerhans, 2008; McGuigan et al., 2003). In sunfish *Lepomis macrochirus* and *L. cyanellus*, the lotic habitat shaped streamlined body, while the lentic environment promoted deeper body in fish (Gaston & Lauer, 2015). In stagnant or low‐velocity‐flow habitats, the deepened body of fish improved foraging capability and predator avoidance (Franssen & Tobler, 2013). The streamlined body shape decreased drag exerting on fish from water and subsequently saved energy (Webb, 1984). However, for southern *H. leucisculus* populations, the relationship between mPC2 and the interaction (cPC1 × cPC2) indicates inland fish are deeper bodied compared with coastal populations. The stagnant and/or low‐flow‐velocity habitats could account for the deeper bodied shape for fish from the populations of Kunming and Qujing, as fish from Kunming population resided in Dianchi Lake and Qujing population resided in a small pond.

Despite the criticisms from several scientists (Legendre & Fortin, 2010; Guillot and Rousset, 2013), the partial Mantel test can be a powerful approach to analyze multivariate data. It is still able to applied, but with the caveat that other analyses are incorporated to guarantee a robust and consistent outcome (Diniz‐Filho et al., 2013). Here, our MANCOVA data render powerful support for partial Mantel test results and can verify the significance between morphological divergence and climatic gradients.

In this study, the weighted evaluation by partial Mantel test indicated significant association between pairwise morphological distances and climatic distances, which confirms that divergent selection pressures among discrepant environments account for the intraspecies morphological diversification (Riesch et al., 2018). However, after controlling the climate distance as a confounding variable with partial Mantel test, we found the pairwise genetic distances are not correlated with morphological distances. It is consistent with the study on damselfish *Pomacentrus coelestis,* which showed no congruence between morphological and genetic structures (Frédérich et al., 2012). So the present study suggests the morphological diversification could be mainly attributable to environmental induced phenotypic plasticity. The neutral marker *cytb* which was used to produce our genetic data is only informative in phylogenic analysis. It is far from sufficient to determine the genetic contribution for the complex morphological variations. To ascertain the definite contribution of heredity or plasticity for the phenotype variations of sharpbelly, cross experiments among individuals of different shape types in common garden will be necessary in future works. In addition, comparative analysis in whole genomes among populations will provide valuable information for the genetic background of morphological diversification (Loh et al., 2008).

## 5 CONCLUSION

The current study mainly describes morphological differentiation for body shape of sharpbelly. The results showed that southern populations were deeper bodied while northern populations were more slender. Inland populations were more streamlined while coastal populations were of deeper body. It predicts that divergent selective pressures are crucial in determining body shape in sharpbelly, while genetic variation has less contribution to morphological differentiation. The formation of Nanling Mountain Range could drive genetic differentiation between Beihai population and those from Yangzi River Basin.

## CONFLICT OF INTEREST

None declared.

## AUTHOR CONTRIBUTIONS

**Lihong Wang:** Conceptualization (equal); data curation (lead); formal analysis (equal); investigation (equal); methodology (lead); project administration (lead); resources (lead); software (lead); validation (lead); visualization (equal); writing–original draft (lead); writing–review and editing (equal). **Long Zhu:** Formal analysis (equal); investigation (equal); methodology (equal); resources (equal). **Kui Tang:** Data curation (equal); investigation (equal); resources (equal); software (equal); validation (equal). **Mengyu Liu:** Formal analysis (equal); investigation (equal); resources (equal); validation (equal). **Xue Xue:** Data curation (equal); resources (equal); software (equal). **Gaoxue Wang:** Conceptualization (equal); formal analysis (equal); investigation (equal); project administration (equal); resources (equal); supervision (equal); writing–original draft (equal); writing–review and editing (equal). **Zaizhao Wang:** Conceptualization (lead); formal analysis (lead); funding acquisition (lead); investigation (equal); resources (lead); supervision (lead); writing–original draft (equal); writing–review and editing (lead).

## Supporting information

Table S1‐S6

## Data Availability

DNA sequences: Genbank accessions MW006099‐MW006463.
