# Supplementary material for: Population genetic structure of sharpbelly Hemiculter leucisculus (Basilesky, 1855) and morphological diversification along climate gradients in China
Source: Ecol Evol. 2021 May 1;11(11):6798–813. doi: 10.1002/ece3.7528 (PMC8207360; doi:10.1002/ece3.7528)
Supplement: Supplementary file 1 — Table S1‐S6 [file ECE3-11-6798-s001.docx]

**Appendix**

**Table S1** Information of sampling sites for eighteen *H. leucisculus* populations

| Population | Location | Province | Coordinates | Drainage |
| --- | --- | --- | --- | --- |
| XA | Xi’an | Shannxi | 34.3285N 109.0521E | Huanghe River |
| QX | Qianxian | Shannxi | 34.5463N 108.0713E | Huanghe River |
| DT | Datong | Shanxi | 39.9871N 113.5385E | Huanghe River |
| TG | Taigu | Shanxi | 37.4316N 112.5842E | Huanghe River |
| LY | Luoyang | Henan | 34.4112N 112.2721E | Huanghe River |
| KF | Kaifeng | Henan | 34.4704N 114.2135E | Huanghe River |
| ZC | Zoucheng | Shandong | 35.5052N 117.1313E | Huanghe River |
| CQ | Chongqing | Chongqing | 29.4124N 105.5833E | Yangzi River |
| WH | Wuhan | Hubei | 30.4790N 114.3750E | Yangzi River |
| TH | Taihu | Zhejiang | 30.9496N 120.1435E | Yangzi River |
| AK | Ankang | Shannxi | 32.5898N 108.8560E | Yangzi River |
| NT | Nantong | Jiangsu | 31.8755N 120.9754 | Yangzi River |
| TL | Tongling | Anhui | 31.0824N 117.8743E | Yangzi River |
| QJ | Qujing | Guizhou | 25.2764N 104.8099E | Yangzi River |
| KM | Kunming | Yunnan | 24.7693N 102.7007E | Yangzi River |
| QT | Qiantang | Zhejiang | 30.0574N 119.9860E | Qiangtang River |
| BH | Beihai | Guangxi | 21.7064N 109.1777E | Nanliujiang River |
| HN | Hainan | Hainan | 19.2279N 108.9498E | Changhuajiang River |

**Table S2** Climatic and geographic parameters of eighteen *H. leucisculus* populations. Climate data from 1981 to 2010 were obtained from Chinese meteorological data network (http://data.cmc.cn). Data of elevation and distance to sea were obtained from Google Earth (http://earth.google.com).

| Population | Elevation (m) | Distance to the sea (Km) | Mean annual  Temperature (°C) | Annual temperature  difference (°C) | Extreme maximum  Temperature (°C) | Extreme minimum temperature (°C) | Annual precipitation (mm) |
| --- | --- | --- | --- | --- | --- | --- | --- |
| XA | 385.0 | 981.3 | 13.1 | 10.6 | 41.7 | -18.6 | 521.9 |
| QX | 624.4 | 1061.2 | 12.9 | 10.2 | 40.2 | -15.8 | 534.8 |
| DT | 1044.0 | 537.3 | 7.3 | 13.2 | 39.2 | -28.1 | 369.3 |
| TG | 791.0 | 566.0 | 10.4 | 13.2 | 40.5 | -22.7 | 397.3 |
| LY | 146.0 | 621.6 | 13.9 | 11.1 | 41.2 | -19.4 | 568.7 |
| KF | 76.0 | 444.0 | 14.6 | 9.8 | 40.7 | -15.0 | 623.0 |
| ZC | 85.0 | 216.7 | 14.9 | 9.1 | 40.1 | -16.1 | 686.5 |
| CQ | 417.0 | 1484.3 | 18.2 | 6.8 | 44.3 | -1.9 | 1156.7 |
| WH | 90.0 | 612.1 | 17.1 | 7.7 | 39.6 | -12.8 | 1316.0 |
| QT | 10.0 | 69.8 | 17.0 | 7.8 | 40.3 | -8.4 | 1438.1 |
| TH | 2.7 | 171.7 | 16.3 | 7.4 | 39.2 | -8.5 | 1303.5 |
| AK | 275.0 | 1128.0 | 15.7 | 9.0 | 41.3 | -9.7 | 824.1 |
| NT | 2.0 | 85.9 | 15.4 | 7.2 | 37.1 | -8.6 | 1119.8 |
| TL | 26.0 | 399.3 | 16.6 | 7.6 | 40.1 | -8.9 | 1392.5 |
| QJ | 1874.1 | 1552.0 | 15.1 | 9.9 | 33.0 | -8.6 | 944.6 |
| KM | 1930.0 | 1609.7 | 15.5 | 10.1 | 31.3 | -7.8 | 979.1 |
| BH | 22.0 | 6.8 | 22.9 | 6.5 | 37.1 | 2.6 | 1775.2 |
| HN | 44.0 | 6.6 | 24.9 | 8.9 | 40.2 | 5.4 | 1658.0 |

**Table S3** *Cytb* sequences of *H. leucisculus* from GenBank

| GenBank Accession No | Location | Basin |
| --- | --- | --- |
| KF020958 | Honghuhe, Hubei | Yangtze River |
| KF021105 | Douchang, Jiangxi | Yangtze River |
| KF021125 | Hukou, Jiangxi | Yangtze River |
| KY292651 | Yiliang, Yunnan | Yangtze River |
| AY089718 | Jiulongjiang, Fujiang | Chiu-lung River |
| AY089716 | Lingjiang, Zhejiang | Ling River |
| AY089717 | Minjiang, Fujian | Min River |
| KY292625 | Hechi, Guangxi | Pearl River |
| KY292574 | Hengxian, Guangxi | Pearl River |
| AY089711 | Qinjiang, Guangxi | Pearl River |
| KY292565 | Bobai, Guangxi | Pearl River |
| AY089709 | Nandujiang, Hainan | Nanhai River |
| AY089710 | Wanquanhe, Hainan | Nanhai River |

**Table S4** The pairwise *F_ST_*s for *H. leucisculus* populations

|  | CQ | QX | DT | XA | TG | WH | LY | ZC | QJ | KM | KF | TL | AK | NT | TH | QT | BH | HN |
| --- | --- | --- | --- | --- | --- | --- | --- | --- | --- | --- | --- | --- | --- | --- | --- | --- | --- | --- |
| CQ | 0.0000 |  |  |  |  |  |  |  |  |  |  |  |  |  |  |  |  |  |
| QX | 0.2917 | 0.0000 |  |  |  |  |  |  |  |  |  |  |  |  |  |  |  |  |
| DT | 0.2788 | 0.0746 | 0.0000 |  |  |  |  |  |  |  |  |  |  |  |  |  |  |  |
| XA | 0.6715 | 0.1942 | 0.3768 | 0.0000 |  |  |  |  |  |  |  |  |  |  |  |  |  |  |
| TG | 0.2627 | 0.0809 | 0.2555 | 0.4677 | 0.0000 |  |  |  |  |  |  |  |  |  |  |  |  |  |
| WH | 0.1424 | 0.3011 | 0.152 | 0.6732 | 0.366 | 0.0000 |  |  |  |  |  |  |  |  |  |  |  |  |
| LY | 0.2984 | 0.0099 | 0.0064 | 0.2345 | 0.1923 | 0.2329 | 0.0000 |  |  |  |  |  |  |  |  |  |  |  |
| ZC | 0.4442 | 0.0929 | 0.1384 | 0.1982 | 0.3091 | 0.4400 | 0.054 | 0.0000 |  |  |  |  |  |  |  |  |  |  |
| QJ | 0.0254 | 0.300 | 0.2786 | 0.6937 | 0.2944 | 0.1429 | 0.2948 | 0.4647 | 0.0000 |  |  |  |  |  |  |  |  |  |
| KM | 0.7534 | 0.7598 | 0.7586 | 0.9143 | 0.7752 | 0.776 | 0.7482 | 0.8118 | 0.7960 | 0.0000 |  |  |  |  |  |  |  |  |
| KF | 0.7043 | 0.7140 | 0.7188 | 0.8648 | 0.7238 | 0.7248 | 0.7086 | 0.7616 | 0.7378 | 0.3158 | 0.0000 |  |  |  |  |  |  |  |
| TL | 0.6732 | 0.6918 | 0.6965 | 0.8237 | 0.7075 | 0.7062 | 0.6928 | 0.7372 | 0.6979 | 0.8593 | 0.8351 | 0.0000 |  |  |  |  |  |  |
| AK | 0.3811 | 0.1650 | 0.2580 | 0.5522 | 0.2549 | 0.4298 | 0.2064 | 0.3371 | 0.3766 | 0.8609 | 0.7867 | 0.7420 | 0.0000 |  |  |  |  |  |
| NT | 0.5150 | 0.4483 | 0.4603 | 0.6731 | 0.5096 | 0.5294 | 0.4481 | 0.5118 | 0.5431 | 0.767 | 0.7276 | 0.323 | 0.5179 | 0.0000 |  |  |  |  |
| TH | 0.1686 | 0.2495 | 0.2846 | 0.5828 | 0.2706 | 0.3030 | 0.2697 | 0.3639 | 0.1668 | 0.7681 | 0.7191 | 0.6838 | 0.2367 | 0.4880 | 0.0000 |  |  |  |
| QT | 0.4354 | 0.4964 | 0.3127 | 0.7960 | 0.5784 | 0.2087 | 0.4060 | 0.5980 | 0.4700 | 0.8388 | 0.7817 | 0.7636 | 0.6451 | 0.6042 | 0.5267 | 0.0000 |  |  |
| BH | 0.7471 | 0.7557 | 0.7611 | 0.8549 | 0.7691 | 0.7599 | 0.7551 | 0.7687 | 0.7662 | 0.7629 | 0.7688 | 0.8279 | 0.7894 | 0.7520 | 0.7765 | 0.7693 | 0.0000 |  |
| HN | 0.8949 | 0.9073 | 0.9050 | 0.9602 | 0.9130 | 0.9049 | 0.9028 | 0.9217 | 0.9117 | 0.9229 | 0.9048 | 0.9238 | 0.9342 | 0.8917 | 0.9068 | 0.9194 | 0.0700 | 0.0000 |

**Table S5** The pairwise morphological Mahalanobis distances among *H. leucisculus* populations

|  | CQ | QX | DT | XA | TG | WH | LY | ZC | QJ | KM | KF | TL | AK | NT | TH | QT | BH | HN |
| --- | --- | --- | --- | --- | --- | --- | --- | --- | --- | --- | --- | --- | --- | --- | --- | --- | --- | --- |
| CQ | 0.000 |  |  |  |  |  |  |  |  |  |  |  |  |  |  |  |  |  |
| QX | 3.823 | 0.000 |  |  |  |  |  |  |  |  |  |  |  |  |  |  |  |  |
| DT | 2.872 | 3.881 | 0.000 |  |  |  |  |  |  |  |  |  |  |  |  |  |  |  |
| XA | 3.410 | 4.296 | 4.166 | 0.000 |  |  |  |  |  |  |  |  |  |  |  |  |  |  |
| TG | 3.618 | 3.831 | 3.855 | 4.213 | 0.000 |  |  |  |  |  |  |  |  |  |  |  |  |  |
| WH | 3.941 | 4.269 | 4.617 | 4.840 | 3.596 | 0.000 |  |  |  |  |  |  |  |  |  |  |  |  |
| LY | 4.606 | 4.495 | 5.066 | 4.244 | 4.409 | 4.489 | 0.000 |  |  |  |  |  |  |  |  |  |  |  |
| ZC | 4.699 | 3.470 | 5.366 | 4.971 | 4.244 | 4.123 | 3.416 | 0.000 |  |  |  |  |  |  |  |  |  |  |
| QJ | 8.258 | 8.084 | 8.179 | 7.162 | 7.510 | 8.308 | 5.782 | 6.685 | 0.000 |  |  |  |  |  |  |  |  |  |
| KM | 7.216 | 8.202 | 6.770 | 8.637 | 8.552 | 8.761 | 8.496 | 9.023 | 11.588 | 0.000 |  |  |  |  |  |  |  |  |
| KF | 6.029 | 6.444 | 6.121 | 6.246 | 6.804 | 6.662 | 4.970 | 6.036 | 7.775 | 5.993 | 0.000 |  |  |  |  |  |  |  |
| TL | 5.509 | 5.105 | 5.207 | 5.934 | 5.598 | 5.810 | 5.690 | 6.046 | 7.608 | 9.058 | 6.723 | 0.000 |  |  |  |  |  |  |
| AK | 6.325 | 5.293 | 5.569 | 6.864 | 5.188 | 5.629 | 6.324 | 5.942 | 8.551 | 8.962 | 7.307 | 4.665 | 0.000 |  |  |  |  |  |
| NT | 6.033 | 5.134 | 5.325 | 6.515 | 5.704 | 6.084 | 6.081 | 6.190 | 9.118 | 7.814 | 6.552 | 4.270 | 3.092 | 0.000 |  |  |  |  |
| TH | 5.971 | 5.830 | 5.394 | 6.741 | 6.140 | 5.660 | 5.690 | 5.408 | 7.547 | 8.733 | 6.674 | 5.810 | 6.005 | 6.418 | 0.000 |  |  |  |
| QT | 3.893 | 4.025 | 3.763 | 5.155 | 4.723 | 3.949 | 4.644 | 4.615 | 8.078 | 7.566 | 5.572 | 4.514 | 5.348 | 5.040 | 3.419 | 0.000 |  |  |
| BH | 5.565 | 5.200 | 5.749 | 6.907 | 5.893 | 5.550 | 5.753 | 4.975 | 8.335 | 8.523 | 7.231 | 6.060 | 6.349 | 6.380 | 6.664 | 5.640 | 0.000 |  |
| HN | 3.986 | 3.954 | 4.552 | 5.406 | 4.816 | 4.439 | 4.387 | 3.989 | 7.898 | 7.708 | 6.396 | 6.089 | 6.371 | 6.230 | 5.942 | 4.460 | 3.581 | 0.000 |

**Table S6** The pairwise climatic Mahalanobis distances among *H. leucisculus* populations

|  | CQ | QX | DT | XA | TG | WH | LY | ZC | QJ | KM | KF | TL | AK | NT | TH | QT | BH | HN |
| --- | --- | --- | --- | --- | --- | --- | --- | --- | --- | --- | --- | --- | --- | --- | --- | --- | --- | --- |
| CQ | 0.000 |  |  |  |  |  |  |  |  |  |  |  |  |  |  |  |  |  |
| QX | 1.431 | 0.000 |  |  |  |  |  |  |  |  |  |  |  |  |  |  |  |  |
| DT | 2.595 | 1.180 | 0.000 |  |  |  |  |  |  |  |  |  |  |  |  |  |  |  |
| XA | 1.615 | 0.502 | 1.067 | 0.000 |  |  |  |  |  |  |  |  |  |  |  |  |  |  |
| TG | 2.238 | 0.881 | 0.427 | 0.656 | 0.000 |  |  |  |  |  |  |  |  |  |  |  |  |  |
| WH | 0.357 | 1.288 | 2.401 | 1.371 | 2.018 | 0.000 |  |  |  |  |  |  |  |  |  |  |  |  |
| LY | 1.663 | 0.802 | 1.247 | 0.322 | 0.819 | 1.369 | 0.000 |  |  |  |  |  |  |  |  |  |  |  |
| ZC | 1.235 | 0.983 | 1.765 | 0.716 | 1.341 | 0.900 | 0.547 | 0.000 |  |  |  |  |  |  |  |  |  |  |
| QJ | 2.572 | 2.467 | 3.157 | 2.969 | 3.131 | 2.811 | 3.259 | 3.243 | 0.000 |  |  |  |  |  |  |  |  |  |
| KM | 2.874 | 2.811 | 3.478 | 3.313 | 3.466 | 3.126 | 3.603 | 3.583 | 0.344 | 0.000 |  |  |  |  |  |  |  |  |
| KF | 1.311 | 0.868 | 1.613 | 0.566 | 1.189 | 0.993 | 0.404 | 0.152 | 3.194 | 3.535 | 0.000 |  |  |  |  |  |  |  |
| TL | 0.471 | 1.499 | 2.581 | 1.531 | 2.185 | 0.237 | 1.485 | 0.972 | 3.015 | 3.325 | 1.090 | 0.000 |  |  |  |  |  |  |
| AK | 0.750 | 0.713 | 1.850 | 0.873 | 1.488 | 0.576 | 0.972 | 0.705 | 2.566 | 2.902 | 0.705 | 0.793 | 0.000 |  |  |  |  |  |
| NT | 0.335 | 1.374 | 2.487 | 1.454 | 2.101 | 0.086 | 1.444 | 0.963 | 2.847 | 3.159 | 1.063 | 0.173 | 0.662 | 0.000 |  |  |  |  |
| TH | 0.513 | 1.524 | 2.597 | 1.543 | 2.198 | 0.275 | 1.488 | 0.969 | 3.059 | 3.368 | 1.090 | 0.044 | 0.821 | 0.215 | 0.000 |  |  |  |
| QT | 0.691 | 1.657 | 2.692 | 1.628 | 2.284 | 0.458 | 1.538 | 0.999 | 3.247 | 3.555 | 1.134 | 0.232 | 0.972 | 0.404 | 0.189 | 0.000 |  |  |
| BH | 1.205 | 2.634 | 3.799 | 2.803 | 3.437 | 1.440 | 2.806 | 2.308 | 3.122 | 3.343 | 2.420 | 1.337 | 1.951 | 1.362 | 1.344 | 1.371 | 0.000 |  |
| HN | 1.042 | 2.455 | 3.592 | 2.561 | 3.209 | 1.192 | 2.527 | 2.007 | 3.278 | 3.527 | 2.130 | 1.042 | 1.748 | 1.108 | 1.040 | 1.029 | 0.401 | 0.000 |
